# *In vitro* synergism of a water insoluble fraction of *Uncaria tomentosa* combined with fluconazole and terbinafine against resistant non-*Candida albicans* isolates

**DOI:** 10.1080/13880209.2016.1242631

**Published:** 2016-12-08

**Authors:** Renata Cougo Moraes, Anderson Ramos Carvalho, Aline Jacobi Dalla Lana, Samuel Kaiser, Bruna Pippi, Alexandre Meneghello Fuentefria, George González Ortega

**Affiliations:** a Post-Graduate Program in Pharmaceutical Sciences (PPGCF), Faculty of Pharmacy, Universidade Federal do Rio Grande do Sul (UFRGS), Porto Alegre, RS, Brazil;; b Post-Graduate Program in Agricultural and Environmental Microbiology (PPGMAA), Universidade Federal do Rio Grande do Sul (UFRGS), Porto Alegre, RS, Brazil

**Keywords:** Natural products, checkerboard, antifungal resistance, candida, polyphenols

## Abstract

**Context:***Uncaria tomentosa* D.C. (Rubiaceae) has several biological activities, including activity against resistant *Candida* strains. The synergistic interaction with terbinafine or fluconazole can be an important alternative to overcome this resistance.

**Objectives:** The potential synergy between a water insoluble fraction (WIF) from *Uncaria tomentosa* bark and the antifungals terbinafine (TRB) and fluconazole (FLZ) against non-*Candida albicans* resistant strains was investigated.

**Materials and methods:** TRB and FLZ, alone and combined with WIF, were tested by the checkerboard procedure using the micro-dilution technique against seven isolates of *Candida glabrata* and *C. krusei*. The molecular interactions occurring outside the cell wall were evaluated by scanning electron microscopy, Fourier transform infrared (FT-IR) and differential scanning calorimetry (DSC) analysis.

**Results:** The checkerboard inhibitory assay demonstrated synergy for WIF:TRB and WIF:FLZ combinations, respectively. The best synergistic cell damage was demonstrated unequivocally for the associations of WIF and TRB (1.95:4.0 μg/mL) and WIF and FLZ (1.95:8.0 μg/mL). The comparison of the FT-IR spectra of the antifungal alone, and in combination with WIF, allows recognizing clear differences in 3000, 1600, 1400, and 700–800 cm^−1^ bands. Additionally, modifications on TRB and FLZ thermograms were clearly noticed after their combination with WIF.

**Conclusions:** DSC and infrared analysis demonstrated intermolecular interactions between WIF and either TRB or FLZ. Hence, quite likely the synergistic effect is related to interaction events occurring outside the cell wall between antifungal and cat’s claw proanthocyanidins. A direct action on the cell wall is suggested, without connection with the ABC efflux pump mechanism.

## Introduction

*Candida* species are harmless saprophyte yeasts, a normal component of the human biota in the gastrointestinal tract and oral and vaginal mucosae. These yeasts can cause superficial infections manifested as thrush and vaginitis; excepting in immune-compromised and immune-suppressed patients, for instance, to whom they can cause severe systemic infections. Risk factors for patients include infection by the human immune deficiency virus (HIV), anti-cancer therapy, organ transplantation, abdominal surgery, catheters, diabetes and the use of broad-spectrum antibiotics (Cruz et al. 2002; Morschhäuser 2002).

In addition, new emerging non-*Candida albicans* (NCA) species and isolates from then even more become a public health challenge due to antifungal resistance. Also, therapeutic approaches are limited by drug-safety, owing the biochemical and metabolic similarities between fungal cell and mammal one (Méan et al. 2008).

Fluconazole (FLZ) and terbinafine (TRB) belong to the main antifungal currently used in the antifungal therapy, which act in different steps of the cell membrane biosynthesis. Triazole derivative FLZ was former the antifungal of choice used in treatment of candidiasis (Bossche 1985). However, its non-rationale use has contributed to the development of resistance in *Candida* spp. (Sanglard 2003). TRB, an allylamine class antifungal, currently is indicated for the treatment of infections caused by dermatophytes. In addition, TRB has shown *in vitro* activity against a wide variety of *Candida* spp. (Ryder et al. 1998), and for the oral treatment of nail candidiasis as well (Segal et al. 1996).

To overcome fungal resistance, the combination of antifungal and non-antifungal drugs represents a successful therapeutic approach, given the multiplicity of fungal targets against which current agents are effective (Mukherjee et al. 2005). It includes effective combinations of FLZ with plant extracts and substances isolated from them (Amber et al. 2010; Endo et al. 2010; Yan et al. 2012). Nonetheless, the association of plant derivatives aiming a synergic effect with TRB for the treatment of candidiasis was not reported until now.

*Uncaria tomentosa* D.C. (Rubiaceae) is a vine widely spread in the South American rainforest and popularly known as cat's claw (Heitzman et al. 2005; Zhang et al. 2015). Many studies have been performed focusing the biological activities of its bark, especially antitumoral (Kaiser et al. 2016), immunostimulant (Montserrat-De La Paz et al. 2015), anti-inflammatory (Aguilar et al. 2002), antiviral (Caon et al. 2014), antioxidant (Pilarski et al. 2006), antimicrobial activities (Heitzman et al. 2005; Ccahuana-Vasquez et al. 2007; Kloucek et al. 2007; Zhang et al. 2015), which frequently have been ascribed to its oxindole alkaloid fraction.

On the other hand, immune modulatory (Lenzi et al. 2013), antioxidant and anti-inflammatory activities (Amaral et al. 2009) were linked to the cat’s claw low molecular weight, and water soluble polyphenols. Concerning the high molecular weight polyphenol fraction from cat’s claw bark there are no scientific reports about any biological activity, as far we know. It is a very low water-soluble fraction, which can fall down during a further extract processing by concentration of aqueous and hydro-ethanolic extracts. Maybe it explains why it has been usually disregarded in biological works.

In this work, we investigated the antifungal activity of the insoluble fraction of polyphenols from cat’s claw bark against NCA species, and its synergy in combination with ﬂuconazole and terbinafine. The involvement possible molecular interactions occurring outside the cell wall is also discussed.

## Materials and methods

### Obtaining of water insoluble fraction (WIF)

An authentic sample of *U. tomentosa* stem barks provided from Peru kindly donated by Induquímica S.A. (Lima, Peru), was comminuted in a cutter mill (SK1 Retsch, Germany), provided with a 2 mm steel sieve. The extraction from cat’s claw bark was performed with hydro-ethanolic solutions 50% (v/v) by 2 h dynamic maceration in magnetic stirring plate (300 rpm) (RO 15 Power, IKA, Germany) at room temperature (23 ± 1 °C) (Kaiser et al. 2013a), followed by filtration and concentration under vacuum at 40 °C up to half of their original weight (BüchiR-114, Germany) aiming to eliminate the ethanol. The concentrated extract was stand overnight in cold (10 ± 1 °C). After, a voluminous precipitated could be verified. For obtaining the water insoluble fraction (WIF), this precipitated was separated from the water soluble fraction by filtration under vacuum and dried in oven (37 °C ± 1 °C) throughout 24 h.

### Chemical characterization of WIF

The complete chemical characterization of WIF was suitably performed in a previous study (Moraes et al. 2015). Briefly, contents and profiles of oxindole alkaloids (Kaiser et al. 2013b), quinovic acid glycosides (Pavei et al. 2012), and low molecular weight polyphenols (Pavei et al. 2010) were carried out by using HPLC-PDA methods previously validated. On the other hand, the proanthocyanidins content was performed by the vanillin acid method (Sun et al. 1998).

### Microorganisms

The set of NCA strains included: *Candida krusei* ATCC 6258, CK01, CK04; *Candida glabrata* CG40039, CG10, RL02, RL03, all of them from the culture collections of the Laboratory of Applied Mycological Research, Universidade Federal do Rio Grande do Sul, Porto Alegre, Brazil.

### Work samples and reference drug solutions

All work samples were dissolved in methanol 2.5% (v/v) to accomplish a concentration of 1.0 mg mL^−1^. TRB (Cristália, Brazil) and FLZ (Cristália, Brazil) were dissolved in ultrapure water (Milli Q, Millipore, Bedford, MA, USA). The solvents used to dissolve samples, the positive control to verify the right inoculum growth, and negative control containing RPMI alone were used as controls.

### Cell growth inhibition

TRB and FLZ, alone and combined with WIF, were tested by the checkerboard procedure using the micro-dilution technique (Kontoyiannis & Lewis 2004). Solutions of the products tested at concentrations determined from their MIC values were used. Aliquots of 50 μL of each work sample were dispensed into a 96-well microplate and mixed with 100 μL of inoculum to get a unique combination of concentrations of drugs and substances previously extracted. The microplates were incubated at 35 °C for 48 h, and the optical density evaluated visually and spectrophotometrically (Envision 2104 Multilaber Reader, Perkin Elmer, USA). The results were expressed in percentage of cellular damage. The assay was performed in quadruplicate. Interactions between antifungals and fractions were determined through the Fractionary Inhibitory Concentration Index (FICI). This index was calculated by the sum of the fractional inhibitory concentrations (FIC) considering the values of the MIC of antifungal in combination and that of antifungal alone (FICI = FIC_A _+_ _FIC_B_). The interaction was defined as synergistic for FICI ≤0.5, as additive for 0.5 < FICI <1, as indifferent for 1 ≤ FICI <4, and as antagonistic for FICI was ≥4 (Lewis et al. 2002; White et al. 1996).

### Cellular damage assay with MTT

The quantitative evaluation of synergism was evaluated by the percentage of cell damage assay, with modifications (Chiou et al. 2001). After the incubation time (3 h), the supernatant from each well was carefully removed and discarded. A 100 μL-aliquot of MTT solution (0.05 mg mL^−1^) was added to each well, followed by incubation for 3 h at 32 °C. Subsequently, the supernatant was removed again and discarded. Separately, 100 μL of isopropanol was added and the cell contents homogenized individually. Next, an aliquot of each well transferred to a new microplate, following the order of pipetting. The absorbance was measured at 570 nm and 690 nm. The cell damage percent was calculated by the Equation 1 – [(A_570nm_ − A_690nm_ with drug)/(A_570nm_ − A_690nm_ without drug)] × 100, where the absorbance with the drug are the data of interest and the absorbance without drugs acts as positive control.

### Obtaining of samples mixture – WIF-TRB and WIF-FLZ

Separately, appropriate amounts of WIF, TRB and FLZ were accurately weighted to attain mixture ratios of 1:4 and 1:2 (w/w) for WIF-TRB and WIF-FLZ, respectively. Each sample was dissolved in methanol 2.5%, magnetically stirred in a water-bath by 48 h, at 32 °C, protected from daylight. After this period, all samples were freeze dried as usually (Edwards EF4 Modulyo freeze dryer, UK) and kept at 4 °C until use.

### Scanning electron microscopy (SEM) of yeasts

*Candida krusei* (CK04) previously treated as described above in Antifungal susceptibility assay, using WIF alone and combined with FLZ and TRB, were fixed with 2.5% glutaraldehyde for 24 h at 10 °C. Post-fixation was carried out with 1% osmium tetroxide in cacodylate buffer for 1 h. Subsequently, the samples were dehydrated in grades acetone, critical-point dried in CO_2_ coated with gold and examined with a scanning electron microscope (AG - EVO 50 Carl Zeiss, Germany) (Maurya et al. 2011). Non-treated yeast was used as positive control.

### Scanning electron microscopy (SEM) of samples mixture

The photomicrographs of the samples were taken using a Scanning electron microscope (JSM 6060, Tokyo, Japan) at a voltage of 10 kV. The samples were previously mounted on aluminium stubs using doubled-sided adhesive tape and vacuum-coated with a thin layer of gold.

### FT-IR analysis of samples mixture

FT-IR spectra from WIF-TRB and WIF-FLZ mixtures were recorded in a frequency range of 4000-600 cm^−1^, using a resolution of 4 cm^−1^ and after 40 accumulations (Shimadzu DR-8001 FTIR spectrometer, Japan). KBr discs were prepared by compressing under vacuum of physical blends of about 1.5 mg each.

### Differential scanning calorimetry (DSC) analysis

Accurately weighed samples of about 2 mg of each WIF-TRB and WIB-FLZ mixtures were analyzed in crimped aluminium pans (Shimadzu DSC-60 calorimeter, Japan). Operating conditions were 10 °C min^−1^ of heating rate (25 to 350 °C) and nitrogen flow at 50 mL min^−1^.

### FT-IR analysis of pseudomycelium

The effect of WIF, TRB and WIF-TRB on the cell wall and cell membrane of the *C. krusei* strain CK04 was assessed using a WIF: antifungal mixture ratio 1:4 (w/w) at a sub-inhibitory concentration of 8:32 μg/mL. Separately, a 100 μL aliquot of each fungal suspension containing 1 × 10^6^ to 5 × 10^6^ CFU mL^−1^ was uniformly applied on Petri dishes containing potato dextrose agar and provided of a central hole of 7 mm in diameter (Galindo et al. 2013), purposely excavated to receive 100 μL of each sample. Milli-Q water (Milli-Q System, Millipore) was used as a control. After incubation by 48 h at 32 °C, a yeast sample was collected by carefully scraping, frozen at once at −20 °C, lyophilized, and properly stored until analysis. This procedure was performed in triplicate.

### Efflux-pump assay

The activity of efflux-pumps was assayed using the inhibitor verapamil, which decreased the MIC when resistance was correlated to the ATP-binding Cassete (ABC) efflux pump mechanism (Prudêncio et al. 2000). Verapamil was directly dissolved into RPMI culture medium with an established final concentration of 0.098 mg mL^−1^ (200 μM). All the strains were tested with and without adding verapamil to the RPMI 1640 medium. Minimal inhibitory concentration (MIC) values were determined by broth micro-dilution using the two-fold dilution method according to the CLSI guidelines (M27-A3) with RPMI-MOPS (RPMI 1640 medium containing l-glutamine and without sodium bicarbonate) (Sigma-Aldrich Co., St Louis, MO) buffered to pH 7.0 with 0.165 M MOPS buffer. The FLZ and TRB concentrations tested ranged from 500 to 0.98 μg mL^−1^ against *C. krusei* and *C. glabrata*. The assay was performed in triplicate.

### Statistical analysis

ANOVA followed by Tukey’s test were applied, and results expressed as mean ± SEM. Differences were considered statistically significant for *p* < .05. Data analyses were performed using Minitab 16.0 software (Minitab Inc., State College, PA).

## Results

### Chemical characterization of WIF

The complete chemical characterization of WIF was performed by Moraes et al. (2015) in a previous study. This fraction showed a high content of proanthocyanidins (70.80%, w/w) derived from cathequin and epicathenin oligomers. Moreover, WIF also showed minor contents of oxindole alkaloids (7.91%, w/w), quinovic acid glycosides (7.79%, w/w), and low molecular weight polyphenols (3.77%, w/w). In relation to oxindole alkaloids profile, WIF was composed only by pentacyclic oxindole alkaloids, mainly, mitraphylline and pteropodine. In addition, the peak 3, a diglycosilated derivative from quinovic acid previously characterized by MS analysis (Pavei et al. 2012), was the major derivative found in WIF.

### Inhibitory studies by the checkerboard assay

The combination of WIF with TRB and FLZ was able to reduce the MIC values determined by the inhibitory assay and either additive or synergic effects could be clearly noticed in all isolates tested. The median FICIs of checkerboard micro-dilution assays for the yeasts studied are shown in Table 1. For WIF-TRB combination a synergistic effect was observed in four different isolates, being two *C. krusei* and two *C. glabrata*, but also for WIF-FLZ in three isolates, being one *C. krusei* and two *C. glabrata* (Table 1). In other isolates an additive effect was observed.

**Table 1.** Fractional inhibitory concentration (FIC) and type of interaction obtained by the checkerboard microdilution assay for testing a combination of antifungal agents.

		TRB + WIF				FLZ + WIF			
	Strains	TRB	WIF	FIC	Interaction	FLZ	WIF	FIC	Interaction
*C. krusei*	CK01	0.500	0.260	0.760	additive	0.250	0.266	0.516	additive
	CK04	0.125	0.133	0.258	synergism	0.250	0.250	0.500	additive
	CK6258	0.062	0.250	0.312	synergism	0.125	0.250	0.375	synergism
*C. glabrata*	CG10	0.500	0.266	0.766	additive	0.250	0.266	0.516	additive
	CG40039	0.062	0.250	0.312	synergism	0.125	0.266	0.391	synergism
	RL03/RL02*	0.062	0.066	0.129	synergism	0.250	0.033	0.283	synergism

WIF: water insoluble fraction from *U. tomentosa*; TRB: terbinafine; FLZ: fluconazole.

* RL03 is corresponding to TRB + WIF and RL02 to FLZ + WIF.

### Cellular damage assay with MTT

The results indicate that TRB and FLZ tested alone up to a 64 μg mL^−1^ concentration was unable to inhibit the growth of any isolated considered (Table 2). The addition of WIF to TRB resulted in enhancement of the antifungal activity of TRB. Thus, TRB and WIF combined in a 8:1.95 μg mL^−1^concentration ratio, respectively, caused higher growth inhibition on the CK6258 terbinafine resistant isolate (ca. 88%) than TRB and WIF alone (40.7% and ca. 20%, respectively). TRB and WIF in a 4:1.95 μg mL^−1^concentration ratio, respectively, caused significant cell damage (79.52%) regarding the CK04 isolate. Terbinafine, the WIF-FLZ was able to induce a significant synergic effect on near all isolates. Regarding the fluconazole resistant isolate CK04, a cell damage of about 80% could be noticed for WIF and FLZ in a concentration ratio of 1.95:8 μg mL^−1^. In that case, the compounds alone showed 50% of cell damage below using the same concentration ratio.

**Table 2.** Percentage of cell damage caused by terbinafine (TRB) and fluconazole (FLZ) alone and their combinations with the water insoluble fraction from *U. tomentosa* bark (WIF), after the checkerboard microdilution method.

		(a) Combination WIF-TRB			(b) Combination WIF-FLZ		
Strains		Comb1	Comb2	Comb3	Comb1	Comb2	Comb3
CK04							
	C (μg/mL)	7.81/64	7.81/32	15.62/32	15.62/16	7.81/16	3.91/16
CDA (%)		45.07^D^/50.23^CD^	45.07^D^/52.51^CD^	52.51^CD^/52.51^CD^	52.51^B^/40.05^C^	45.07^BC^/40.05^C^	35.80^C^/40.05^C^
CDC (%)		90.05^AB^	91.22^A^*	70.01^BC^	89.52^A^	82.67^A^*	73.13^A^
CK01							
	C (μg/mL)	7.81/4	3.91/4	1.95/4	7.81/8	3.91/8	1.95/8
CDA (%)		26.02^B^/15.52^B^	24.54^B^/15.52^B^	14.69^B^/15.52^B^	26.02^B^/32.49^B^	24.54^B^/32.49^B^	14.69^B^/32.49^B^
CDC (%)		57.56^A^	72.42^A^	79.52^A^*	80.22^A^*	73.27^A^	73.96^A^
CK6258							
	C (μg/mL)	1.95/4	1.95/8	1.95/16	7.81/16	3.91/16	3.91/32
CDA (%)		19.83^C^/18.38^C^	19.83^C^/40.74^B^	19.83^C^/37.92^BC^	70.40^C^/12.61^E^	49.68^D^/12.61^E^	49.68^D^/13.04^E^
CDC (%)		85.16^A^*	87.95^A^	86.4^A^	91.36^A^	79.15^B^	94.15^A^*
CG10							
	C (μg/mL)	15.62/32	3.91/32	0.98/64	15.62/16	7.81/16	3.91/16
CDA (%)		51.02^CD^/57.12^BCD^	42.92^DE^/57.12^BCD^	16.89^E^/55.01^CD^	51.02^BC^/39.58^D^	40.51^D^/39.58^D^	42.92^CD^/39.58^D^
CDC (%)		88.89^A^	75.74 ^ABC^*	82.73^AB^	55.08^B^	86.77^A^*	52.67^BC^
CG40039							
	C (μg/mL)	3.91/4	1.95/4	1.95/8	3.91/8	1.95/8	0.98/8
CDA (%)		35.26^B^/33.77^B^	32.86^B^/33.77^B^	32.86^B^/37.85^B^	35.26^B^/31.59^B^	32.86^B^/31.59^B^	28.20^B^/31.59^B^
CDC (%)		67.54^A^	70.25^A^*	87.65^A^	62.89^A^	64.57^A^	60.79^A^*
RL03/RL02							
	C (μg/mL)	1.95/8	0.98/8	0.98/4	3.91/8	1.95/8	0.98/32
CDA (%)		22.88^BC^/31.37^B^	7.32^D^/31.37^B^	7.32^D^/11.62^CD^	30.22^C^/16.77^D^	30.19^C^/16.77^D^	24.02^CD^/40.84^B^
CDC (%)		61.16^A^	65.35^A^	56.92^A^*	44.03^B^	24.55^CD^	65.00^A^*

WIF: water insoluble fraction from *U. tomentosa*; TRB: terbinafine; FLZ: fluconazole; antifungal cell damage alone (CDA) and in combination (CDC) expressed in percentage (%). Over cellular damage is shown concentrations (C) of the respective antifungal agents. Three combinations with additive and synergistic effects were chosen to demonstrate the interaction.

* Combinations used for FIC calculating.

** Different letters represents results statistically distinct (*p* < .05) for MICs.

### Scanning electron microscopy (SEM) of yeasts

**Figure 1.**
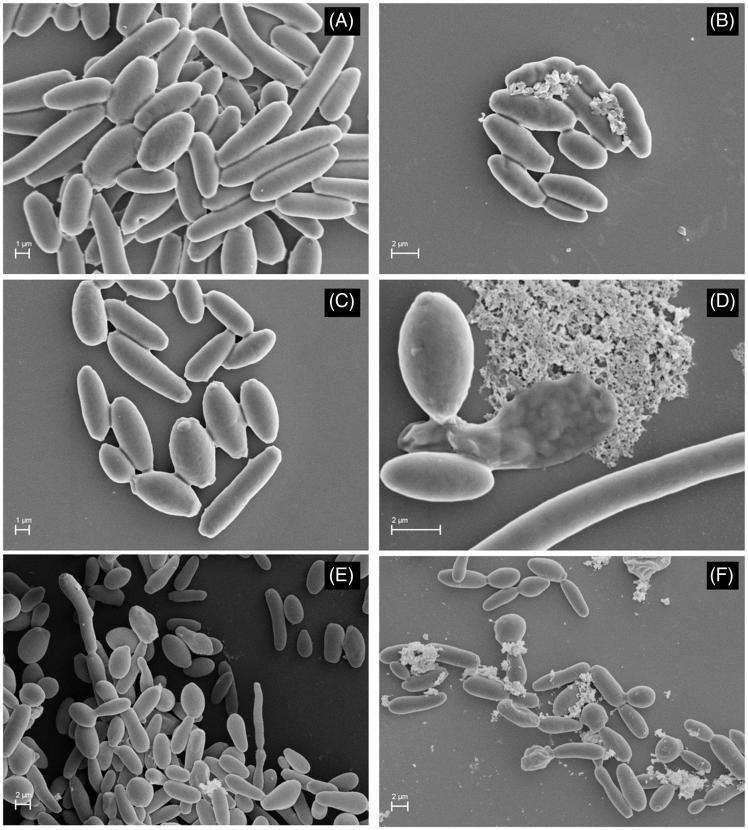
SEM-micrographs of Candida krusei (CK04) treated with fraction WIF from *Uncaria tomentosa*, alone and in combination with terbinafine (TRB) and fluconazole (FLZ). (A) Control without treatment (10,000×); (B) WIF 7.81 μg ml-1 (10,000×); (C) FLZ resistant strain 64 μg ml-1 (10,000×); (D) WIF-FLZ 3.91: 16 μg ml-1concentration ratio (18,000×); (E) TRB resistant strain 32 μg ml-1 (5000×); (F) WIF-TRB 7.81: 16 μg ml-1 (1000×).

The effect of WIF, WIF-TRB and WIF-FLZ on morphology and ultra-structure of resistant isolates was exemplified by *C. krusei* CK04 (Figure 1). The normal budding profile (Figures 1(A),(C) and (E)). Contrasted with the irregular budding patterns, material deposited on the cell walls and loss of cell integrity, after treatment with WIF alone (Figure 1(B)) and it combined with TRB (Figure 1(D)) and FLZ (Figure 1(F)).

### Scanning electron microscopy (SEM) of samples mixture

**Figure 2.**
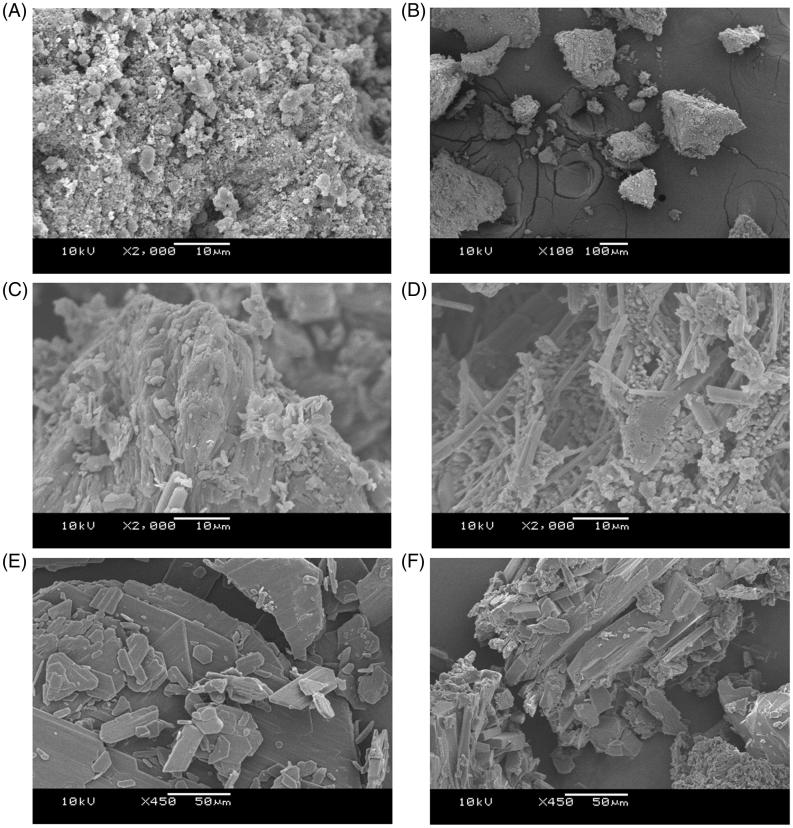
SEM photomicrographs obtained from (A) and (B) water insoluble fraction of *U. tomentosa* (WIF), (C) fluconazole, (D) freeze-dried WIF-fluconazole mixture, (E) terbinafine, (F) freeze-dried WIF-terbinafine association.

The photomicrographs from WIF, FLZ, WIF-FLZ, TRB and WIF-TRB (Figure 2) allow observing definite crystal modifications unambiguously. FLZ micrographs showed agglomerated particles having irregular and cracked surface, and occasionally acicular habit (Figures 2(A) and (B)), while the TRB ones presented flat plate-shaped crystals of varied sizes (Figure 2(E)). Changes in the crystal habitat of FLZ were noticed after analysis of the FLZ: WIF mixture (Figure 2(D)), specifically, the appearance of needle shaped micro-crystals imbibed in an amorphous and loose matrix. The WIF-TRB mixture occurred as broken plates with a slightly irregular surface and amorphous structures around it (Figure 2(F)). At first view, these morphologic changing in the crystal habitat can be attributed to factors as freeze drying and crystallization medium itself, but that does not completely exclude the possibility of molecular interactions as the cause of these modifications.

### Differential scanning calorimetry (DSC) analysis

**Figure 3.**
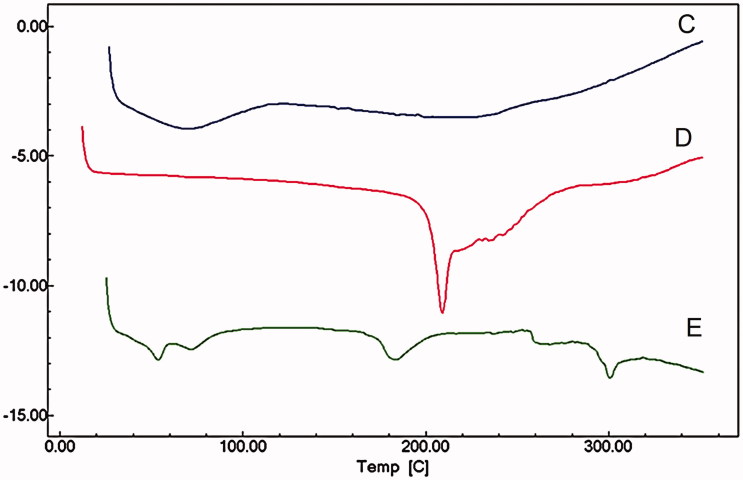
The differential scanning calorimetry (DSC) curves of (A) water insoluble fraction from *U. tomentosa* bark (WIF), (B) fluconazole (FLZ), (C) WIF:FLZ 1:2 combination ratio, (D) terbinafine (TRB), (E) WIF:TRB 1:4 combination ratio.

DSC thermograms of WIF, FLZ, TRB and the corresponding combinations are in Figure 3. WIF presents a broad and undefined endothermic peak at about 80 °C, revealing its amorphous state (Figure 3(A)). FLZ thermogram showed a single, sharp peak at 140 °C and a broad endothermic process centred at about 300 °C, reflecting thermal degradation (Figure 3(B)). The TRB thermogram showed a characteristic clear onset peak at 205 °C followed with a broad endothermic process. The effect of the combination of both TRB and FLZ with WIF was exemplified using critical concentration ratios (Figures 1(D) and (F)). Modifications on TRB and FLZ thermograms were clearly noticed after their combination with WIF. The complete disappearance of TRB peak and the displacement of the FLZ onset peak to 175 °C indicated a strong molecular interaction of WIF with TRB and FLZ indistinctly. Moreover, further DSC analysis showed that the intensity of the molecular interaction is depending on the antifungal: WIF concentration ratio (omitted data).

### FT-IR analyses of the WIF from *U. tomentosa*, terbinafine, fluconazole, and their combinations

**Figure 4.**
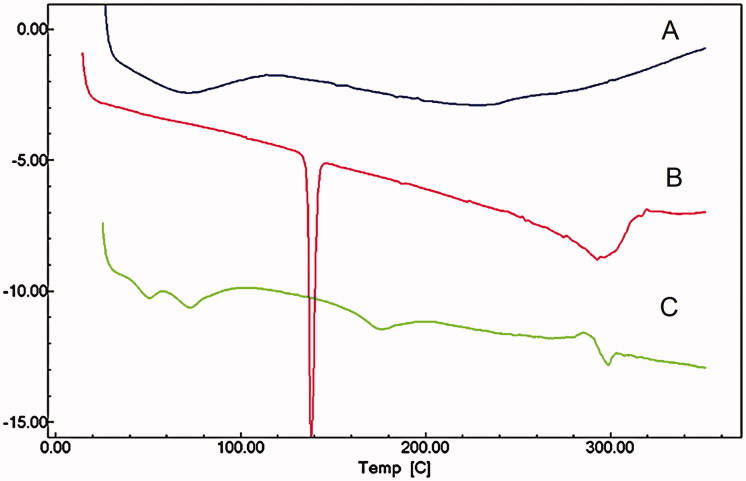
(A) FT-IR spectrum of WIF fraction; (B) terbinafine; (C) complex (WIF:TRB in 1:4 combination ratio); (D) WIF; (E) fluconazole; (F) association (WIF:FLU in 1:2 combination ratio).

The FT-IR spectrum from WIF showed a strong and wide band at 3230 cm^−1^ (assigned to a OH polymer association), 1613 cm^−1^ (ring C–C stretch of phenyl characteristic in polyphenol compounds), 1445 cm^−1^ (aromatic C = C), and 1065 cm^−1^ (ring aromatic C–H bond) (Figures 4(A) and (D)). By comparing to literature data (Foo 1981), some bands suggested the presence of catechins and procyanidin derivatives, for instance, bands at 1540–-1520 cm^−1^ (related to hydroxyl of catechins) and the fingerprint bands at 780–770 cm^−1^ (out-of-plane deformation aromatic H–bonds).

The TRB spectrum (Figure 3(B)) showed a large intensity band in 2440 cm^−1^ (C≡C vibration), which associated with the additional bands 1450–1500 cm^−1^ (aromatic C = C) and 600-800 cm^−1^ (C–Cl) allowed a reliable identification of terbinafine hydrochloride (Silverstein et al. 1991; Arunprasad et al. 2010). As a tertiary amine, no typical bands of N–H bond are present in the spectrum range of 3300–3500 cm^−1^.

The FLZ spectrum was comparable to literature data (Cyr et al. 1996), especially the bands at 3119, 1507 and 1420 cm^−1^ (triazole CH bonds), 1618 cm^−1^ (aromatic ring stretching), 1074 cm^−1^ (C–OH bond) and 1114 cm^−1^ (C–F bond).

The FT-IR spectra of WIF-TRB combination were illustrated by the WIF:TRB mixture in 1:4 concentration ratio. The main modification appraised were the complete disappearance of the WIF spectrum bands at 3308 and 1613 cm^−1^, and the TRB band former located at 2440 cm^−1^ (assigned to a C≡C vibration). Regarding the last one, also the formation of a new band at 2631 cm^−1^ was noticed. TRB alone showed three bands in the 1360–1470 cm^−1^ region, which was reduced to just one located at 1453 cm^−1^. Additional spectral changes could be noticed in the 700–900 cm^−1^ region.

The FT-IR spectra of WIF-FLZ combination was exemplified by the mixture with a 1:2 concentration ratio. The main modification were the disappearance of 3308 cm^−1^ and 3119 cm^−1^ bands of WIF and FLZ, respectively; the appearance of a new band in the 2600–2900 cm^−1^ range and an intensity decrease of 1614 cm^−1^ band, referring of the aromatic ring carbon bonds. Also, the 1300–1400 cm^−1^ and 600–800 cm^−1^ range evidenced some alterations.

### FT-IR analysis of the pseudomycelium after treatment with the insoluble fraction combined to TRB

**Figure 5.**
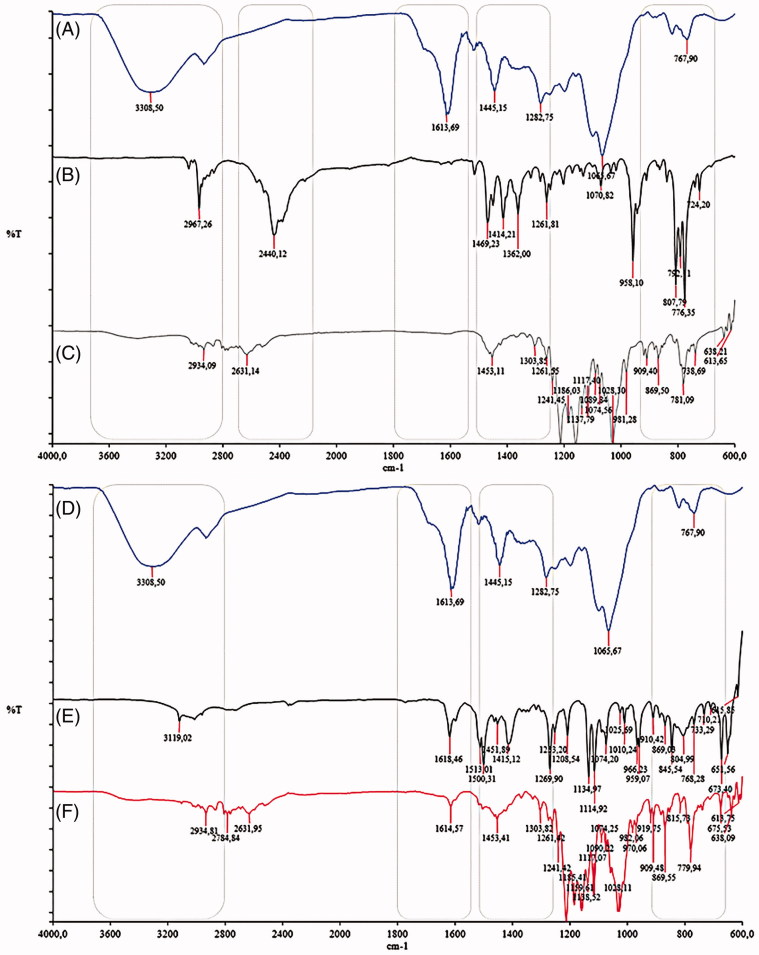
FT-IR spectra of *C. krusei* pseudomycelium exposed to (A) control without treatment, (B) water insoluble fraction (WIF) from *U. tomentosa* bark, (C) terbinafine resistant strain, (D) WIF:TRB in 1:4 combination ratio.

FT-IR spectra of the pseudomycelium of *C. krusei* showed tiny differences after treatment with WIF, either alone or combined with TRB (Figure 5). After treatment with WIF, no substantial change was observed in the region of 2996–2800 cm^−1^ and 1500–1200 cm^−1^, whose absorption bands are associated to the membrane lipids, as earlier described (Galindo et al. 2013). Minor changing induced by WIF-TRB in the 1359 cm^−1^ band was tentatively assigned to interactions with the cell wall lipids. In contrast, more intense spectral changes in 1600–400 cm^−1^ and 900–600 cm^−1^ regions suggested a molecular interaction of WIF with the cell wall proteins and polysaccharides (Figure 4), but different from those observed with TRB alone (Figure 4(C)). The FT-IR spectrum of WIF-FLZ retained the same features described in those spectra of FLZ and WIF alone (data not shown).

### Efflux-pump assay

The activity of efflux-pumps was checked using the inhibitor verapamil, which decreases the MIC when the resistance is due to ATP-binding Cassete (ABC) efflux pump. The fungal susceptibility of two multiresistant isolates, namely, CK6258 and CG40039, was tested for TRB and FLZ. In both the cases, no substantial changing of the MIC values could be detected.

## Discussion

The combination of antifungal drugs with vegetable compounds has been referred as a potential strategy to improve antifungal activity via additive and synergistic effects (Wagner & Ulrich-Merzenich 2009). Regarding plant polyphenols, very few works had focused this issue. Worth mentioning is the combination of polyphenols from *Punica granatum* with fluconazole, which allowed a two-fold decreasing of the MIC values against *C. albicans* and *C. parapsilosis* strains (10). Even though the synergic effect was ascribed to the polyphenol punicalagin, no further evidence was presented to enlighten better the mechanism behind this finding. Similarly, several studies have focused on showing the antifungal activity of plant derivatives (Silva et al. 2011; Ahmad et al. 2013; Jun et al. 2013); however, few have approached the impact on the cell morphology of chemical and physicochemical interactions that happen between these compounds.

The chemical characterization of WIF was performed in a previous study (Moraes et al. 2015). This water insoluble fraction from cat’s claw bark comprises mainly proanthocyanidins (70.80%, w/w) and, in a minor extend, oxindole alkaloids (7.91%, w/w), quinovic acid glycosides (7.79%, w/w) and low molecular weight polyphenols (3.77%, w/w). Also, the anti-*Candida* activity of WIF was previously established (MICs from 3.90 to 15.62 μg/mL against *C. glabrata*, *C. krusei* and *C. parapsilosis*) (Moraes et al. 2015). However, its potential synergism with FLZ and TRB received little attention at that time. Essentially, the oxindole alkaloids (OALK), low molecular mass polyphenols (LMPOL), and quinovic acid derivatives (QAD) contents in the water-soluble and water-insoluble fractions from the hydroethanolic extract of cat’s claw bark were earlier assayed by HPLC-PDA. Similar analysis of the insoluble proanthocyanidins (PROAN) fraction showing mainly catechin and epicatechin oligomers (data omitted), has confirmed previous work (Gonçalves et al. 2005) In addition, the content of PROAN was determined in both fractions by the vanillin test (Moraes, et al. 2015). These authors also reported a close relationship between the antifungal activity against *Candida* species and some features related to the chemical composition of both fractions. Thus, they contained significant amounts of OALK, LMPOL and QAD, however, the PROAN content surpass the other ones and was clearly higher in the water-insoluble fraction the in the water-soluble ones (70.80% versus 21.07%). It seemed related to the fact PROAN fraction showed the highest antifungal activity against *Candida,* which even exceeded that determined for terbinafine and fluconazole. Meanwhile OALK, LMPOL, and QAD fractions were rather inactive against both species when tested separately.

In this work, six isolates of *C. krusei* and *C. glabrata* showing azole- and terbinafine-resistance were chosen for this intent, and separately treated with FLZ and TRB, but also by combining them with WIF. In all isolates tested the WIF-antifungal combination proved to be more effective than TRB and FLZ alone. In fact, the fractional inhibitory concentration (FIC) data (Table 1) revealed synergic and additive effects associated to the combination of both antifungals with WIF. Data from the cell damage test allowed reinforcing this finding (Table 2). The synergistic effect was prevalent in those cases where the WIF-TRB combination was tested, specifically, in 66% of the tests. An analogous effect was observed with regard to the WIF-FLZ combination, but here it drops to 50%. Moreover, *C. krusei* isolates were the most susceptible ones, particularly to TRB-WIF combination, where inhibition levels above 80% were noticed. In that case, MIC value of TRB-WIF decreased up to 16-times when compared to TRB alone.Even though antifungal susceptibility was diverse among the *Candida* isolates, some important modifications in cell morphology can be noticed by SEM-analysis. In this occasion, *C. krusei* CK04 was selected for illustration purpose, owing the synergistic effect observed unmistakably for this isolate. SEM-micrographs of CK04 pseudo hyphae treated with sub-inhibitory concentration of WIF-TRB and WIF-FLZ (Figure 1) revealed major morphological alterations, as irregular budding pattern, material deposited on the cell wall, and loss of cell integrity. Through expanding the line of thought to the other isolates, we may assume that both antifungal and synergistic effects may be directly related to damage to the structure of the cell wall, at least. In addition, similar findings reported in literature about alterations on the yeast cell wall ultrastructure, and caused by antifungals associated with plant extracts (Nakamura et al. 2004; Ishida et al. 2006; Endo et al. 2010), tend to corroborate our assumption.

Molecular interactions related to antifungals in association with vegetable products can be easily appraised by DSC (Pawar et al. 2012). In this study the alterations observed in thermograms recorded for the combinations of WIF with terbinafine and fluconazole are distinguish by disappearance of the characteristic endothermic peaks of TRB and the FLZ at 205 °C and 140 °C, as well as the manifestation of new peaks in 195 °C and 175 °C, respectively. Likewise, Al-Marzouqi et al. (2009) studying the fluconazole-β cyclodextrin systems were able to corroborated inclusion complexes by means of the shifting of the fluconazole peaks measured by DSC. Hence, the findings leave little doubt, about molecular interactions taking place between WIF and both drugs.

The FT-IR analysis of WIF revealed the predominance of catechin derivatives and some absorption patterns characteristic of tannins, as previously noticed (Morais et al. 1999; Hye et al. 2009). Besides, it allowed excluding low molecular weight polyphenols from WIF composition, at least in tangible amounts, and also detecting functional groups involved in the interactions between WIF and both antifungals TRB and FLZ. Given that no absorption band near to 1750 cm^−1^ (vibrational C = O stretch or COOH usually related to carboxylic acid (Manrique & Lajolo 2002) was observed, gallic and chlorogenic acid and their derivatives could be excluded from the WIF composition. In addition, by UV-analysis of the methanolic solution of WIF, flavonoids was detected just as traces, as no absorption above 360 nm was detect (data not shown) (Geiger & Quinn 1975).

Tannins are polymeric polyphenols able to complexation of alkaloids through formation of covalent bounds (Spencer et al. 1988) and, presumably other weak bases, as TRB and FLZ. As results, the water-solubility of alkaloids can be improved. In that context, the FT-IR technique allows a straightforward characterization of molecular interactions between polymer-antifungal, or its absence, as earlier related for TRB and the polymer PEG 6000 (Kuminek et al. 2013).

The analysis of WIF, TRB and FLZ by FT-IR and DSC consistently support the hypothesis of molecular interactions between polyphenols and antifungal taking place outside the cell membrane. So, the comparison of the FT-IR spectra of the antifungal alone, and in combination with WIF, allows recognizing clear differences in 3000, 1600, 1400 and 700–800 cm^−1^ bands. It reflects, therefore, molecular interactions between the both antifungal drugs and the WIF fraction from cat's claw bark.

Also, SEM-analysis of TRB and FLZ separately, and combined with WIF, provided a supplementary information supporting the occurrence of antifungal:WIF interaction. The changing in crystal habitat became evident in photomicrographs from TRB alone and combined with WIF, namely, from a plated shape with a smooth surface crystals (Figure 2(E)) to polyhedral blocks having trace of amorphous masses (Figure 2(F)). Similarly, the FLZ crystalline habitat underwent a clear modification from irregular forms (Figure 2(C)) to a more organized needle-shaped form (Figure 2(D)) after mixing with WIF.

The modifications in the microstructure habitat are in agreement with the DSC data described above, when thermal changes in the melting point and other endothermic processes unquestionably occurred after mixing TRB and FLZ with WIF (Figure 3). Despite polymorphism was already recognized in some azole derivatives, as miconazole (Pedersen et al. 1993), at this point a such statement in favour of polymorphism would be questionable, once the analysis was performed using physical mixtures instead of isolated substances. Of note, the crystal habitat of some antifungals, including TRB and FLZ, can undergo also a marked modification according to the crystallization medium and technological processing, but without implying a genuine polymorphism (Pedersen et al. 1993; Kuminek et al. 2013). Consequently, polymorphic events should be discarded in favour of a concrete antifungal-WIF interaction, as proved by FT-IR and DSC data

The FT-IR analysis of the pseudomicelium of *C. krusei* (CK04) substantiated the WIF action on the cell wall. Through the treatment with WIF and WIF-TRB combination, the alteration of the IR bands located at 1600–400 cm^−1^ and 900–600 cm^−1^ denotes modifications in the protein and polysaccharide located in the wall membrane, which were indiscernible in the untreated fungal sample. According to Galindo et al. (2013) a decrease or change in the content of these components may be related to the loss of cell integrity an enhancement of the membrane permeability to antifungal.

Verapamil is a known inhibitor of the efflux pump and has been often used to study the reversion of the resistance to antifungal. In spite of great homology between the amino acid sequence of glycoprotein-P (P-gp) and the fungal ATP-binding cassette (ABC) protein transporters, a great variability in sensitivity of yeast ABC proteins was earlier reported (Sanglard et al. 1997; Sanglard & Odds 2002). Nonetheless, data from the assay performed with FLZ and TRB showed that the resistance of the TRB and FLZ-multi-resistant isolates CK6258 and CG40039 seems to be unrelated with the ABC efflux pump (Guinea et al. 2006).

## Conclusion

A cat's claw water insoluble fraction presents synergistic effect with FLZ and TRB against *C. krusei* and *C. glabrata*. DSC and FT-IR data showed that this fraction mainly composed by proanthocyanidins interacts with FLZ and TERB unquestionable. The participation of polymorphic events and the ABC efflux pump seems to be out of question. The antifungal activity is explained on the basis of molecular interaction outside the cell wall, the issue is relevant and, at the same time challenging.
